# Electromagnetic Real Time Navigation in the Region of the Posterior Pelvic Ring: An Experimental *In-Vitro* Feasibility Study and Comparison of Image Guided Techniques

**DOI:** 10.1371/journal.pone.0148199

**Published:** 2016-02-10

**Authors:** Miguel Pishnamaz, Christoph Wilkmann, Hong-Sik Na, Jochen Pfeffer, Christoph Hänisch, Max Janssen, Philipp Bruners, Philipp Kobbe, Frank Hildebrand, Thomas Schmitz-Rode, Hans-Christoph Pape

**Affiliations:** 1 University of Aachen Medical Center, Department of Orthopedic Trauma, Aachen, Germany; 2 University of Aachen Medical Center, Department of Diagnostic and Interventional Radiology, Aachen, Germany; 3 Helmholtz Institute of RWTH Aachen University & Hospital, Institute of Applied Medical Engineering, Aachen, Germany; 4 Helmholtz Institute of RWTH Aachen University & Hospital, Chair of Medical Engineering, Aachen, Germany; Klinikum rechts der Isar - Technical University Munich - TUM, GERMANY

## Abstract

**Background:**

Electromagnetic tracking is a relatively new technique that allows real time navigation in the absence of radiation. The aim of this study was to prove the feasibility of this technique for the treatment of posterior pelvic ring fractures and to compare the results with established image guided procedures.

**Methods:**

Tests were performed in pelvic specimens (Sawbones^®^) with standardized sacral fractures (Type Denis I or II). A gel matrix simulated the operative approach and a cover was used to disable visual control. The electromagnetic setup was performed by using a custom made carbon reference plate and a prototype stainless steel K-wire with an integrated sensor coil. Four different test series were performed: Group OCT: Optical navigation using preoperative CT-scans; group O3D: Optical navigation using intraoperative 3-D-fluoroscopy; group Fluoro: Conventional 2-D-fluoroscopy; group EMT: Electromagnetic navigation combined with a preoperative Dyna-CT. Accuracy of screw placement was analyzed by standardized postoperative CT-scan for each specimen. Operation time and intraoperative radiation exposure for the surgeon was documented. All data was analyzed using SPSS (Version 20, 76 Chicago, IL, USA). Statistical significance was defined as p< 0.05.

**Results:**

160 iliosacral screws were placed (40 per group). EMT resulted in a significantly higher incidence of optimal screw placement (EMT: 36/40) compared to the groups Fluoro (30/40; p< 0.05) and OCT (31/40; p< 0.05). Results between EMT and O3D were comparable (O3D: 37/40; n.s.). Also, the operation time was comparable between groups EMT and O3D (EMT 7.62 min vs. O3D 7.98 min; n.s.), while the surgical time was significantly shorter compared to the Fluoro group (10.69 min; p< 0.001) and the OCT group (13.3 min; p< 0.001).

**Conclusion:**

Electromagnetic guided iliosacral screw placement is a feasible procedure. In our experimental setup, this method was associated with improved accuracy of screw placement and shorter operation time when compared with the conventional fluoroscopy guided technique and compared to the optical navigation using preoperative CT-scans. Further studies are necessary to rule out drawbacks of this technique regarding ferromagnetic objects.

## Introduction

Percutaneous iliosacral screw placement represents the standard of care in the majority of posterior pelvic injuries [1,2]. However, the incidence of screw misplacement has been reported to be between 2% and 15% [3–6]. Screw malpositioning can result in significant neurological and vascular damage. Intraoperative navigation could diminish the rate of iliosacral screw misplacement [5,7–11].

The accuracy of minimally invasive stabilization techniques has been significantly improved by the implementation of image guided technologies [5,8,9,11,12]. In addition, optical tracking systems have been established [10,11]. However, these are surface based and the required usage of flexible instruments may not be applicable in case of navigation over long distances. Another major disadvantage of this method is its susceptibility to masking or shielding of the cameras by instruments or the surgeon himself (line of sight effect) [13–17]. “Electromagnetic Tracking” (EMT) offers a method that allows real time navigation also in subjacent parts of the body. Furthermore, it avoids any radiation during the surgical procedure [13,18]. However, potential interference factors on the electromagnetic field avoided the widespread clinical use of this technique for a long time [13,15,16,18,19]. New technologies caused an improvement of these drawbacks. Also, automatic error detection and the necessity of smaller electromagnetic fields offer new options for the application [15,16].

The purpose of this study was to examine the use of electromagnetic tracking during minimally invasive screw placement in the treatment of sacral fractures and to compare this methodology to the commonly applied techniques, either under solid fluoroscopy or with additional optical tracking guidance.

## Material and Methods

**Fig 1:**
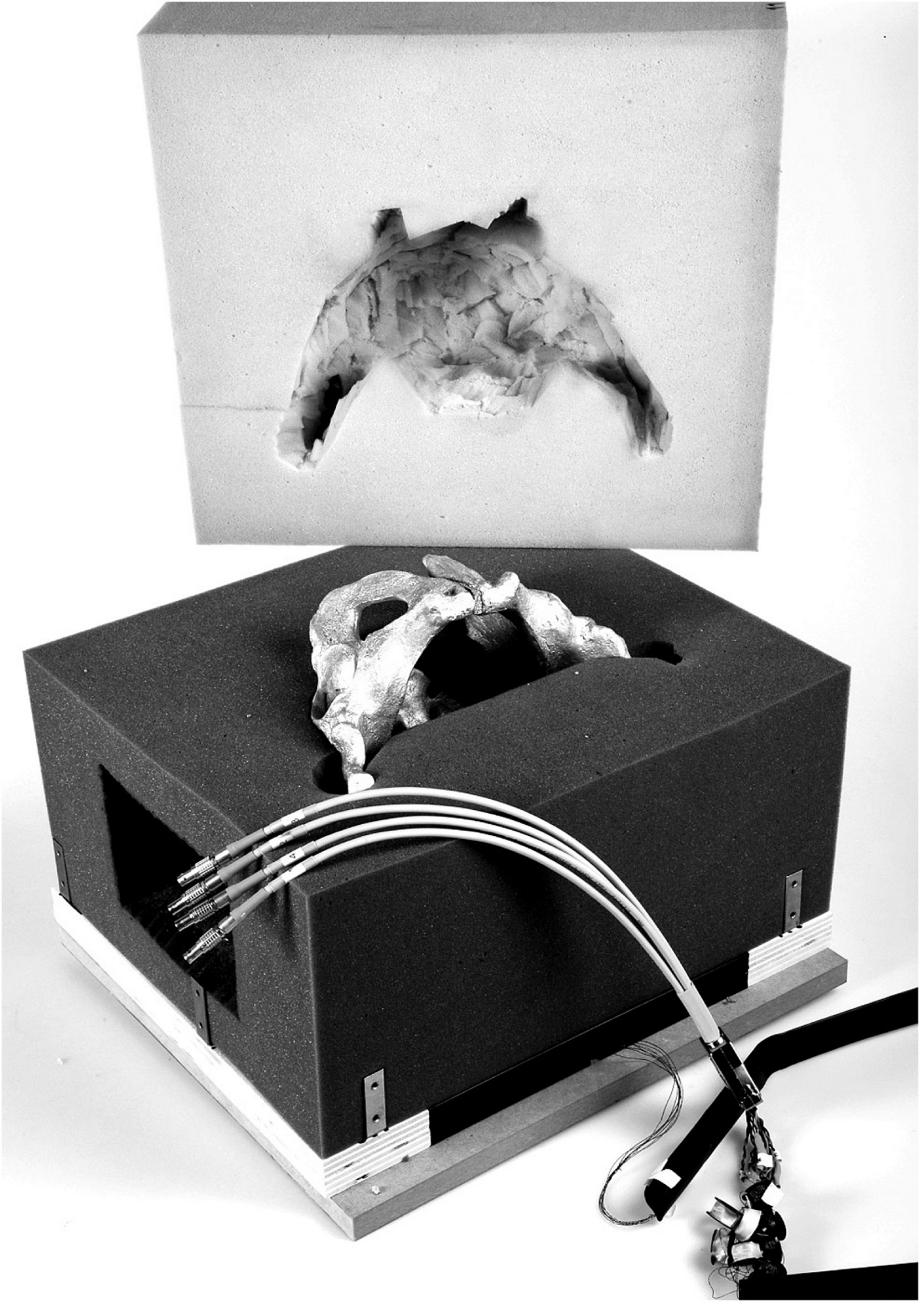
Pelvic model. Illustration of the pelvic model.

Custom made anthropomorphic pelvic specimens (Sawbones^®^) were used. A standardized osteotomy of the sacrum was performed prior to the experiment to create either extraforaminal (Type Denis 1) or transforaminal (Type Denis 2) sacral fractures. The sawbones were embedded in foam cubes with corresponding covers to prevent visual control. Lateral windows on both sides of the cube were substituted by a gel wax matrix to facilitate the operative approach. In case of electromagnetic navigation, the registration panel was fixed in a rack below the pelvic model. The phantom was placed corresponding to a supine position (Fig 1).

**Fig 2:**
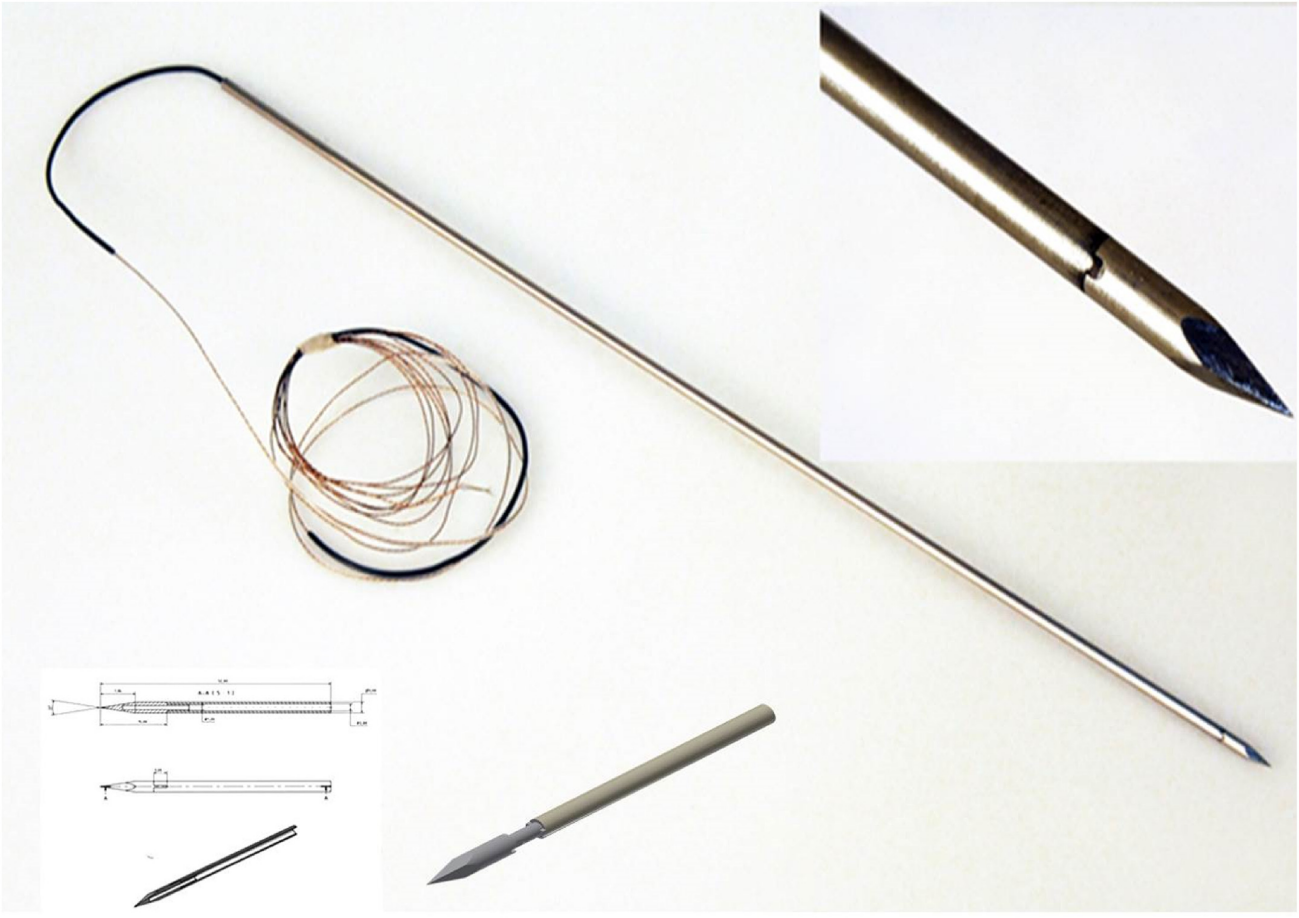
K-wire. Illustration of the electromagnetic K-wire with an integrated sensor coil.

For all fractures, canulated 7.0 mm stainless steel, self-drilling, half thread screws (manufactured by: Pioneer^™^ Surgical, Marquette, USA; distributed by: Zimmer Inc., Warsaw, USA) and corresponding 3mm x 28cm k-wires (Depuy Synthes^®^) were used. Within the electromagnetic group, a custom made K-wire with an integrated EMT coil in the tip of the wire was designed (Fig 2). K-wire positioning was performed by using a standard battery-driven drilling machine (Colibri System, Depuy Synthes^®^).

Overall, four different test setups were performed.

### 1. Optical navigation by preoperative CT-scans (Group OCT)

**Fig 3:**
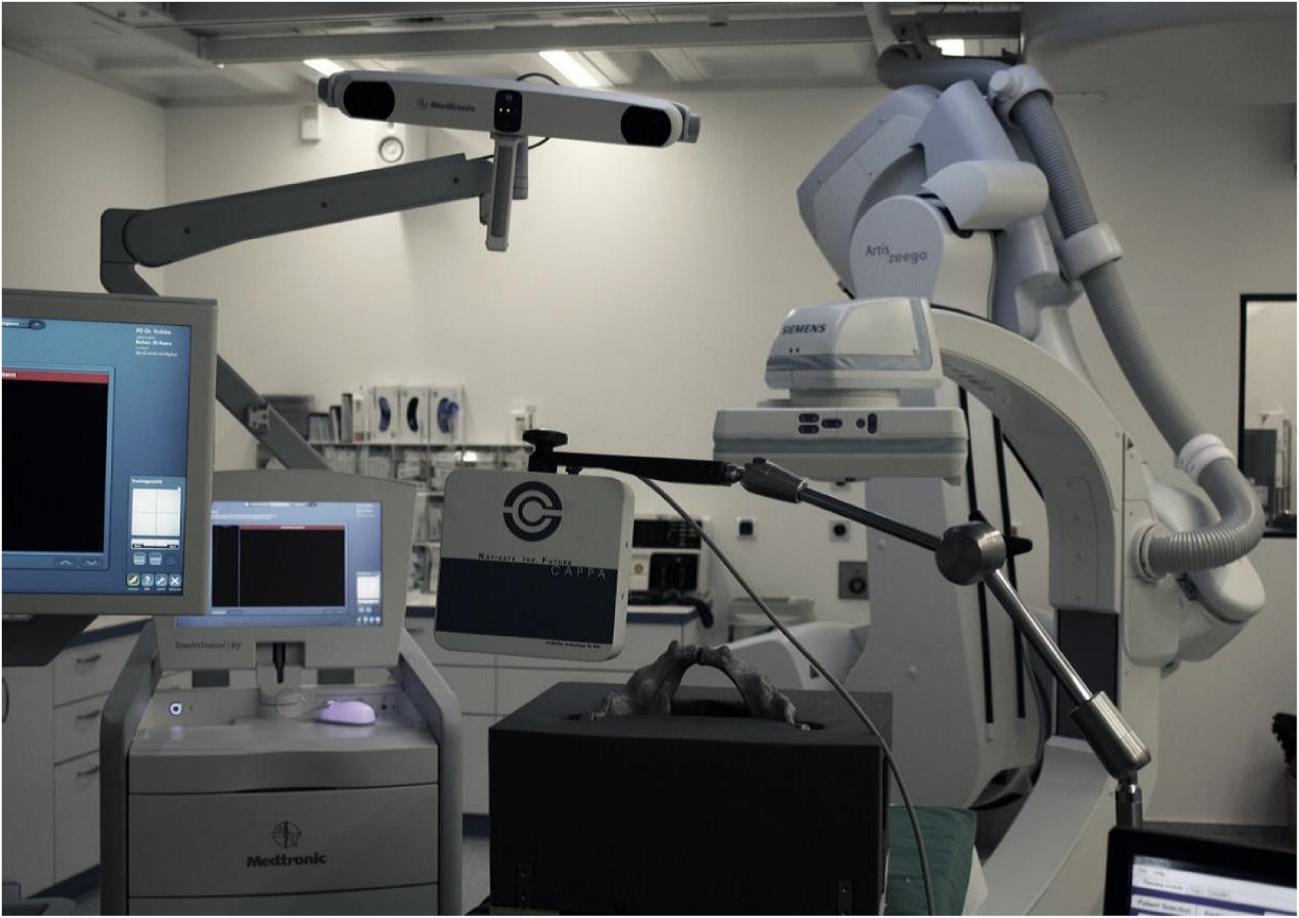
Navigation setup. Illustration of the navigation setup.

**Fig 4:**
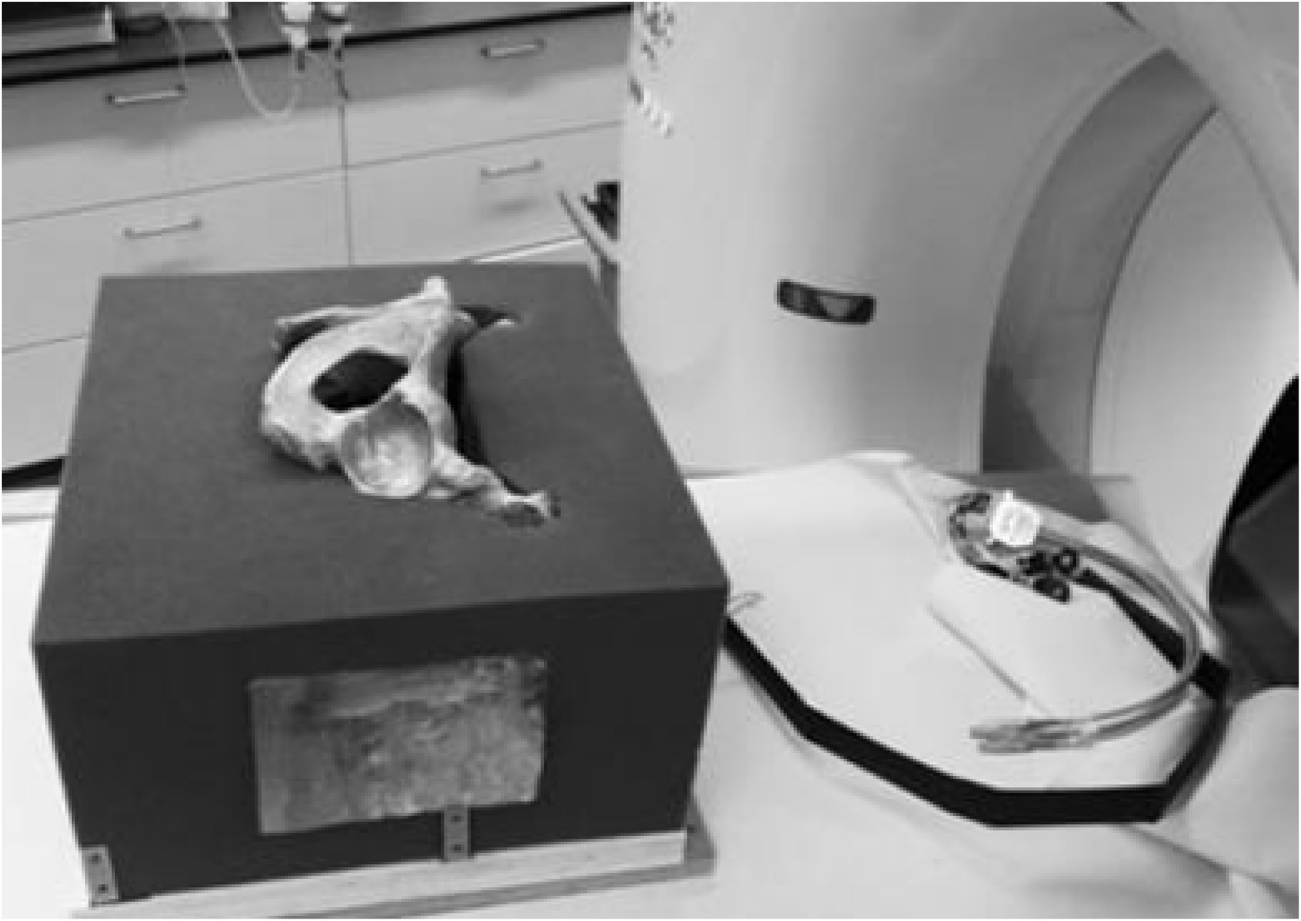
CT-scan. Illustration of the pelvic model during the preoperative CT-scan.

CT-scans (Siemens SOMATOM^®^) of the pelvic models were performed prior to the interventions. The data was secondary transformed to the Navigation System (Stealth Station S7^®^ Surgical Navigation System, Medtronic Inc., Minneapolis, Minnesota, USA) (OCT- and O3D group). Image registration was performed by surface matching. Therefore the cover of the bracket was removed and superficial bone areas were registered precisely (Figs 3 and 4).

### 2. Optical navigation by intraoperative 3-D-fluoroscopy (Group O3D)

The reference frame was placed and connected on the iliac crest by a clamp. Subsequently, a mobile C-arm system (Ziehm Vision FD Vario 3D, Ziehm Imaging GmbH, Nürnberg, Germany) offering 2D fluoroscopy and 3D CT-like multi-planar reconstructions (MPR), was used to acquire the 3D navigation image-set and for intra-interventional fluoroscopy. The image data was directly transferred to the Stealth Station to perform optical navigation. Within both optical navigation setups (O3D and OCT) the k-wire was inserted by the use of a navigated guide sleeve.

### 3. Conventional 2-D-fluoroscopy (Fluoro-Gr)

Within this group screw placement was performed without navigation and solely by intraoperative 2-D fluoroscopic control (System: Ziehm Vision FD Vario 3D; Ziehm Imaging GmbH, Nürnberg, Germany). As described in previous literature the entry point was first justified in the lateral view. Therefore, exact lateral positioning of the image intensifier to the sacrum was necessary. The entry point into the first sacral vertebra was placed next to the sacral canal. The orientation of the k-wire was then turned to the center of the vertebral body. Subsequent the precise k-wire position was verified in the a-p, inlet and outlet view [20]. Central placement of the S2 screw was intended between the sacral foramina S1 and S2.

### 4. Electromagnetic navigation (EMT-Gr)

**Fig 5:**
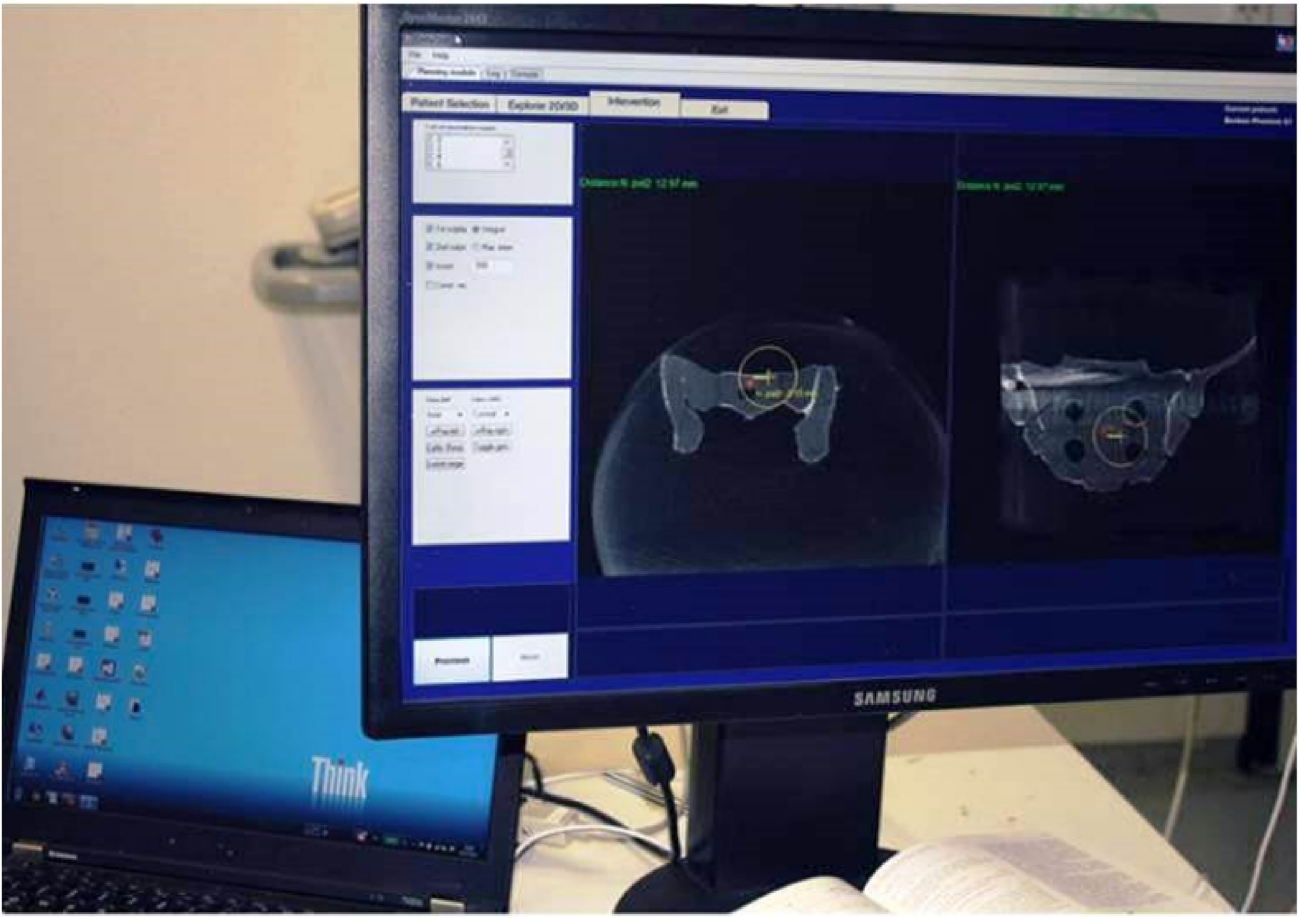
EM navigation. Illustration of the EM navigation software during k-wire positioning.

For the treatments performed under electromagnetic tracking guidance, an Artis zeego (Siemens AG, Forchheim, Germany), a robot-arm mounted C-arm system capable of 2D fluoroscopy and 3D Dyna-CT imaging, was utilized to generate the navigation image set. As a navigation platform, a state-of-the-art electromagnetic tracking system (Aurora^®^, Northern Digital Inc., Waterloo, Ontario, Canada) (Fig 3) was used in combination with a customized navigation software (OrthoTAIX, SurgiTAIX AG, Herzogenrath, Germany). The software implements the Aurora^®^ application programming interface (API) for system control and data acquisition and offers real-time navigation in previously assessed 3D image datasets using MPR views (Fig 5). Image registration was done with a custom made reference plate embedding multiple reference sensor coils and radiopaque markers which can be segmented semi-automatically by the software.

In order to track the wire electromagnetically, a customized boring wire with an embedded sensor coil was designed and built from scratch while trying to adopt the basic characteristics of the K-wires used in the comparative methodologies concerning diameter, strength and resilience. The wires were made from stainless steel type 1.4571 and had a size of the shaft of 3 mm x 0.8 mm x 350 mm (diameter, material thickness and length respectively) and a tip with 3 mm thick solid material (Fig 2). The sensor coil (Aurora, Northern Digital Inc., Waterloo, Ontario, Canada) was fixed to the tip with Loctite M-31CL medical approved glue (Henkel AG & Co. KGaA, Düsseldorf, Germany) and the shaft and tip were merged with epoxy resin type L20 (R&G Faserverbundwerkstoffe GmbH, Waldenbuch, Germany). During the manufacturing process, special care was taken to ensure a precise placement of the sensor coil in the wire in order to minimize static errors. Reference testing was applied for the manufactured prototypes to ensure precise knowledge of, and if necessary correct for, these static errors.

During the CT scan the reference plate was placed below the patient. This reference plate allowed the registration of the image space and electromagnetic space. During this step, the immobilization of the patient was essential. After imaging the gantry was moved out of the operating area and the field generator was positioned accurately to the intervention area. Once the image transfer was completed, the electromagnetic space and the imaging space were coupled together. From this moment the sensor coil in the tip of the K-wire was visualized punctiform within the image data set.

Each group underwent a standardized procedure. The fracture reduction was performed prior to the experiment.

### Definition of terms

1. Tests were started after the radiological verification of the “skin incision” (gel wax); 2. the lateral cortex of the ilium was palpated; 3. the k-wire entry point was justified either by x-ray control or by real time navigation; 4. subsequent, the k-wire was drilled to the sacral midline; 5. cannulated drilling; 6. screw positioning (100mm); 7. removal of the wire; 8. final x-ray control.

The duration of procedure (from the incision to the final screw position) as well as the duration of intraoperative radiation was documented.

To describe the precision of each technique, the screw position accuracy was categorized into four different grades: grade 0: no perforation; grade 1: perforation< 2mm; grade 2: perforation from 2–4mm; grade 3:≥ 4mm perforation. Furthermore the angulation of the screw to the adjacent endplate was measured and the number of k-wire perforations was counted.

All specimens received a CT examination before and after the experimental intervention (Dual-source CT system; Somatom Definition, Siemens, Forchheim, Germany; CT protocol: 64×0.6 mm collimation, 120 kV, 165 mAseff. Axial image data sets (1- mm slice thickness, 0.6-mm increment)) to verify the fracture elements and to analyze screw positioning.

### Statistics

All data was analyzed using SPSS (Version 20, 76 Chicago, IL, USA). Descriptive data are summarized as absolute frequencies and percentages for categorical variables, and delivered as means and standard deviations (SD) for continuous variables. All data were tested for normal distribution. Continuous variables were compared using Student’s t-test in case of approximately normally distributed data or the Mann-Whitney U-test otherwise. Statistical significance was defined as p < 0.05.

## Results

### Feasibility of electromagnetic guidance

In our experimental setup, the reference signal of the sensor coil within the electromagnetic field was constant during the test execution. Neither the use of stainless steel k-wires nor the utilization of the drill led to a signal interruption during the test series. Initial problems were overheating of the wire tip and cable breakages at the exit point from the k-wire. These were eliminated by modifying the prototype prior to the experiment and by the performance of oscillating drilling during the tests.

### Analysis of accuracy

#### Iliosacral screw positioning

**Fig 6:**
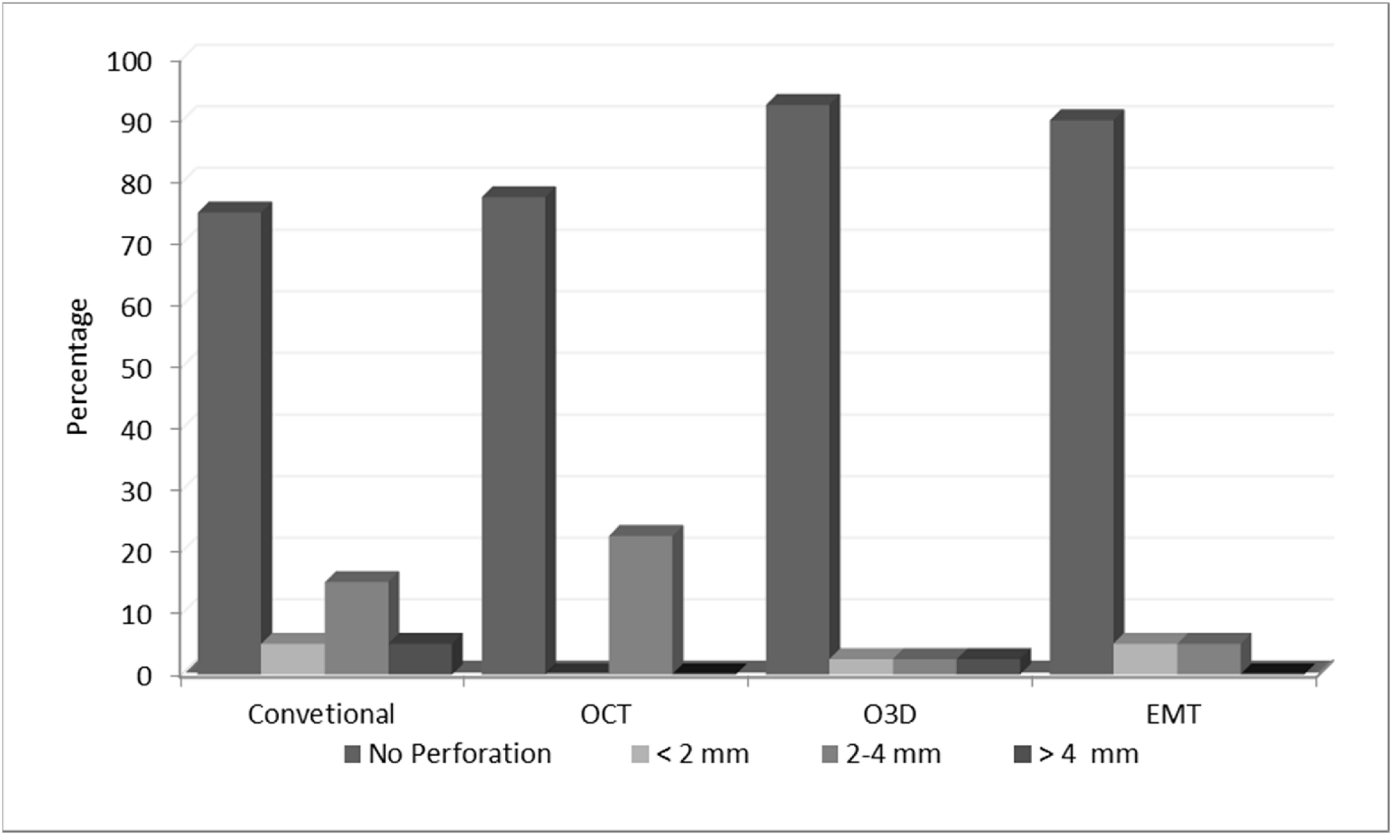
Accuracy of iliosacral screw positioning. Illustration of the accuracy of iliosacral screw positioning depending on the utilized technique.

Overall, 160 iliosacral screws were placed using a pelvic specimen. Optimal screw placement was significantly more frequent in the EMT group (36/40) compared to the groups Fluoro (30/40; p< 0.05) and OCT (31/40; p< 0.05). However, results between EMT and O3D were comparable (O3D: 37/40; n.s.) (Table 1 and Fig 6). Almost all misplacements were observed in screws placed into the second vertebral body (S1: 2/80 vs S2: 24/80; p< 0.001). The fracture type (Denis I or II) did not influence the accuracy of screw positioning in any group (Overall: Misplacements/ Denis 1: 12/81 vs Denis 2: 14/79; n.s.).

**Table 1:** Screw placement accuracy.

	EMT		O3D		OCT		Conventional	
Screw Position	Abs.	%	Abs.	%	Abs.	%	Abs.	%
**Optimal**	36	90	37	92,5	31	77,5	29	75
**< 2 mm**	2	5	1	2,5	0	0	3	5
**2–4 mm**	2	5	1	2,5	9	22,5	6	15
**>4 mm**	0	0	1	2,5	0	0	2	5

#### Screw angulation

The angulation of the screws to the adjacent endplate in the coronary view was similar in all groups (Fluoro: 4°; OCT 3.8°; EMT 3.9°; O3D: 5°; n.s.). Furthermore there was no difference between S1 and S2 screws (S1/2: Fluoro: 4.2°/3.8° n.s.; OCT 3.9°/3.8° n.s.; EMT 3.5°/4.2° n.s.; O3D: 5.3°/4.7°; n.s.).

#### K-wire perforations

Pelvic specimens showed eight K-wire perforations of the cortical bone in the OCT group, six in the Fluoro group and two perforations in the group O3D. No perforations were found in the EMT group. All observed perforations affected the S1 or S2 neuroforamen.

### Duration of intervention

The shortest duration of screw placement into the first sacral vertebra was found in the EMT and the O3D group (EMT 6.26 ±2.11 min vs. O3D 6.59 ±2.43 min; n.s.). The duration was significantly shorter compared to the Fluoro (9.42 min ±3.11; p< 0.001) and the OCT group (11.13 ±5.20 min; p <0.001).

Comparable results were found for screw placement into the second sacral vertebra. The groups EMT and O3D showed comparable results (EMT 8.98 ±3.73 min; O3D 9.37 ±4.69min; n.s.), whereas the duration of the intervention in groups Fluoro (11.96 ±5.17 min; p< 0.001) and OCT (15.47 ±5.67 min; p< 0.001) was significantly longer.

Across all groups, placement of S1 screws (sagittal target range: 630 mm^2^) was achieved significantly faster when compared with S2 screws (sagittal target range: 185 mm^2^) (Overall S1: 8.35 ±3.93 min vs S2 11.45 ±5.44 min; p< 0.05).

### Radiation exposure

Within the OCT and the EMT group, a CT-scan was performed prior to the experiment. The use of different CT units between each setup caused a mean dose-length product (DLP) of 134 mGy*cm within the EMT and 106.3 mGy*cm within the OCT group. This corresponds to an effective radiation dose of 2.546 mSv in the EMT group and 2.0197 mSv in the OCT group, considering the corporality of the pelvic model. Within the O3D group the mean duration of the intraoperative scan was 70 seconds accordingly the dose area product (DAP) was 16.1 cGym* cm^2^.

During the procedure no intraoperative imaging was performed in the electromagnetic group to evaluate the accuracy of the real time navigation. Within the other groups, the O3D group needed significantly less additional intraoperative fluoroscopy (1.2 ±1.36 sec) in comparison to the group OCT (15.4 ±10.48 sec; p< 0.001) which in turn needed significantly less fluoroscopy than the Fluoro group (42.6 ±17.43 sec; p< 0.001).

## Discussion

Optical navigation represents daily routine in many trauma and spine units [12]. However, practical difficulties, such as the line of sight effect and the missing possibility of real-time navigation have been a concern [14]. Electromagnetic tracking is a technique that might improve these issues. New systems offer improved resistance to ferromagnetic objects and might help to establish this technique in the clinical practice [17].

Previous clinical and experimental studies have shown that electromagnetic navigation represents a reliable technique for interventional procedures like radiofrequency ablations of tumors, liver biopsies or punctures of the lumbar facet joints [19,21–23] [24]. However, different factors avoided the widespread use of this technique in trauma surgery [25–27]. Firstly, concerns about interfering ferromagnetic objects within the clinical environment have been raised. In this context, Yaniv et al. reported about differences in the accuracy of the electromagnetic navigation depending on the location of the procedure [28]. Secondly, other studies reported about tracking errors induced by the distance of the CT system to the electromagnetic field [29,30]. In our study different modifications seem to overcome these problems. The integration of an EM sensor coil into the stainless steel K-wire tip and the use of a standard drill avoided an interference of the electromagnetic field. Furthermore, possible interference by the CT gantry and the surgical table were eliminated by the use of a robot-arm mounted 3D Dyna-CT that automatically moved far away from the interventional setup after imaging and the application of a standard carbon spine table. These modifications also appear to be easily implemented in clinical use. Even though not all operating theaters are equipped with a CT unit, the use of a mobile 3-D image intensifier might be generally possible.

In this line, our results can be summarized as follows:

1. Real time navigation of Iliosacral screws with electromagnetic guidance is a feasible procedure
2. Electromagnetic navigation ensures highly accurate screw positioning in the posterior pelvic ring
3. The application of EMT can reduce the operation time and decreases the exposure to radiation for the surgical team during the operation

### Limitations of our study

This is an experimental study and the transferability to the clinical practice is limited. The results of surgical navigation are dependent on the frequency of their application. Within our study no learning curve was found, but we feel that this was caused by the relatively low number of implemented screws considering the different types of navigation. Furthermore, an in vivo study is necessary to investigate the application of the electromagnetic technique more precisely.

Within this study different imaging modalities were used, that may reduce the comparability considering the radiation exposure between the respective groups. On the other hand, the use of pelvic specimens offers similar conditions with regard to the comparability of the applied techniques. Whether this technique is also applicable in patients with internal fixations or prosthesis close to the electromagnetic field cannot be answered by our study and needs to be investigated in further studies. In contrast, by excluding this potential interfering factors the applicability of electromagnetic tracing under “standard conditions” could be analyzed more precisely.

Due to multiple factors (e.g. complex anatomy; difficult intraoperative visulazation), percutaneous iliosacral screw positioning is well known to be technically demanding. Therefore, an overall incidence of screw misplacement between 2% and 15% has been reported [3,5]. Despite the fact that the use of navigation seems to result in more accurate screw positioning [5,11,12] it must be considered that data of optical navigation systems is calculated form the surface of the navigated-object and reflectors of the surgical instruments. Especially in the region of the posterior pelvic ring, the distance between the guide wire and the surface can be more than 10 cm. This poses the risk of bending of the wire and inaccuracies within the navigation process. From this point of view the advantages of EMT seem to be obvious, because this technique is based on real time navigation.

Ricci et al. found in his experimental setup comparable results regarding the accuracy of electromagnetic and optical navigated guidewires [14]. Hoffmann et al. postulates that the use of electromagnetic guidance in scaphoid fractures is superior to the standard fluoroscopic technique [24]. Other studies confirm these results and show a high accuracy of EM-tracking particularly during long distance interventional setups [13,15,16,18,19,21,22].

These studies are in line with our findings and show that electromagnetic guidance can improve the precision of iliosacral screw placement. In our experimental setup we used a custom made guide wire with a cable link at the end. This prototype did not facilitate rotational drilling. Furthermore, the software used in the study just offered the punctate visualization of the sensor coil and a defined target point. Advances of these technical instruments might lead to further improvement regarding the accuracy of EM-tracking in this area. Besides, the quality of imaging, the tracking system, the surgical instruments, the patients’ localizer and the image registration are potential interference factors within the navigation process. Therefore, it is essential to optimize all inaccuracies of all involved components to achieve the most precise operative result.

Several studies showed that electromagnetic navigation reduces the need for intraoperative radiation [13,15,16,18,19,24–26]. But it has to be distinguished between the exposure for the surgical team and the patient. Since the operating theatre will be left during 3D-imagng, the radiation exposure for the surgical team will be at least reduced in all navigation setups compared to the fluoroscopic guided technique. This underlines our findings. Additional intraoperative imaging was not necessary in the EMT group in our study. Within the other test series in our study, the O3D group also facilitated a significant reduced intraoperative radiation. In contrast, within the OCT group intraoperative imaging was frequently necessary (15.4 ±10.48 sec). This was caused by inaccuracies during the surface matching process. The advantage of surface matching is that high quality preoperative imaging can be used. Particularly in obese patients intraoperative 3-D imaging is difficult and usually remains with poor visualization [31]. Even so, we feel that surface matching alone is insufficient for the treatment of posterior pelvic injuries, because the target area is too large resulting in imprecise navigation. Besides, the patients transport from the CT-scan gantry to the operating room may cause alterations of the anatomical conditions by displacing the fractured bone. Particularly in highly unstable fractures thus may lead to inaccuracies within the navigation process.

## Conclusion

Electromagnetic guided iliosacral screw placement is a feasible procedure. This method was associated with improved screw placement accuracy, shorter time of operative procedure and less intraoperative radiation exposure for the surgeon compared to the conventional fluoroscopy guided technique without navigation and compared to the optical navigation using preoperative CT-scans.

## Supporting Information

S1 Table Electromagnetic Navigation Dataset A. (XLSX)
